# Increased sucrose levels mediate selective mRNA translation in Arabidopsis

**DOI:** 10.1186/s12870-014-0306-3

**Published:** 2014-11-18

**Authors:** Magdalena Gamm, Alessia Peviani, Anne Honsel, Berend Snel, Sjef Smeekens, Johannes Hanson

**Affiliations:** Molecular Plant Physiology, Institute of Environmental Biology, Utrecht University, Utrecht, The Netherlands; Theoretical Biology and Bioinformatics, Department of Biology, Faculty of Science, Utrecht University, Utrecht, The Netherlands; Umeå Plant Science Centre, Department of Plant Physiology, Umeå University, SE-90187 Umeå, Sweden

**Keywords:** Arabidopsis, Protein synthesis, Sugar signaling, Translational regulation

## Abstract

**Background:**

Protein synthesis is a highly energy demanding process and is regulated according to cellular energy levels. Light and sugar availability affect mRNA translation in plant cells but the specific roles of these factors remain unclear. In this study, sucrose was applied to Arabidopsis seedlings kept in the light or in the dark, in order to distinguish sucrose and light effects on transcription and translation. These were studied using microarray analysis of steady-state mRNA and mRNA bound to translating ribosomes.

**Results:**

Steady-state mRNA levels were affected differently by sucrose in the light and in the dark but general translation increased to a similar extent in both conditions. For a majority of the transcripts changes of the transcript levels were followed by changes in polysomal mRNA levels. However, for 243 mRNAs, a change in polysomal occupancy (defined as polysomal levels related to steady-state levels of the mRNA) was observed after sucrose treatment in the light, but not in the dark condition. Many of these mRNAs are annotated as encoding ribosomal proteins, supporting specific translational regulation of this group of transcripts. Unexpectedly, the numbers of ribosomes bound to each mRNA decreased for mRNAs with increased polysomal occupancy.

**Conclusions:**

Our results suggest that sucrose regulate translation of these 243 mRNAs specifically in the light, through a novel regulatory mechanism. Our data shows that increased polysomal occupancy is not necessarily leading to more ribosomes per transcript, suggesting a mechanism of translational induction not solely dependent on increased translation initiation rates.

**Electronic supplementary material:**

The online version of this article (doi:10.1186/s12870-014-0306-3) contains supplementary material, which is available to authorized users.

## Background

Plant growth and development depend on energy provided by carbohydrates and the coordination of its storage and mobilization. Energy-consuming processes must be controlled to coordinate growth with energy availability in an ever-changing environment. One of the most energy-demanding cellular processes is protein synthesis: energy has to be provided for amino acid and tRNA synthesis, peptide bond formation, and the biogenesis of the translational machinery [1]. In Arabidopsis rosettes, 10% of the proteins were estimated to be ribosomal proteins, but these numbers are likely higher in rapidly growing tissues [2]. In addition, a large number of non-ribosomal proteins are needed for initiation, elongation, termination, and ribosome recycling during mRNA translation [3]. Translation is considered to be regulated at initiation [4], while elongation and termination rates are proposed to be less affected in most conditions [5]. Initiation rates control the loading of the mRNA into polysomes, affecting translational efficiency [6,7]. How specific mRNAs are selected for translation is poorly understood. A number of mRNA features have been proposed to be involved in translational regulation, including 5′UTR length and nucleotide sequence composition, secondary structure, the presence of uORFs, as well as different cis-acting motifs within the mRNA sequence [8,9].

Several studies address the adaptation of translational efficiency to changes in environmental conditions like mild dehydration [8], hypoxia [9,10], salt and high temperature [11,12]. Stress conditions generally affect translation by decreasing both polysomal occupancy (fraction of an mRNA present in polysomes) and ribosome density (number of ribosomes per mRNA). Interestingly, some mRNAs escape this general stress mediated reduction of translation and maintain or increase polysomal occupancy, ensuring the production of proteins necessary for adaptation. Such translational control is essential for the plant’s response to stress and adds another layer to the regulation of gene expression [13,14]. Translation of other mRNAs is strongly repressed under stress conditions, while their steady-state transcript levels do not change [15]. Recently, some of these mRNAs were found to associate with the UBP1 RNA-binding protein during hypoxia [16]. UBP1 might be involved in sequestering mRNAs in cytoplasmic granules for the duration of the stress and their release for translation during recovery, thus providing a mechanism of translational regulation [16]. Interestingly, the formation of these UBP1-granules might be connected to ATP availability [16], suggesting a link between energy availability and translation. Energy availability has been implicated to affect protein synthesis at several levels [1,17–19].

Increased sucrose availability correlates with increased association of mRNAs to polysomes during daytime, whereas during the night protein synthesis is adapted to limit energy consumption [1,18]. Sucrose starvation of cultured Arabidopsis cells represses translation of most mRNAs, generally independent of changes in steady-state mRNA levels [17]. Similarly, low sugar status induced by unexpected dark treatment of Arabidopsis seedlings leads to a reduction of translation, with mRNAs being sequestered and re-initiated rapidly after re-illumination [19]. Interestingly, many of the mRNAs affected by varying sugar levels encode ribosomal proteins.

Ribosomal protein genes are among the main targets of translational control in many experiments, for example after growth stimulation of germinated maize embryos [20]. Recently, mRNAs encoding ribosomal proteins were proposed to form a ‘regulon’ of coordinated translational regulation [14], likely dependent on the initiation factor eIF3h and the ribosomal protein RPL24B [21]. In *Arabidopsis thaliana*, ribosomal proteins are encoded by more than 240 genes [22,23] of which most are translated to proteins as judged by proteomic experiments [23–25]. Generally, only a single protein of each of the 81 protein families is present in the functional ribosome leading to a great number of possible combinations [26]. A role of the resulting ribosomal heterogeneity is yet to be identified, but mutants lacking specific paralogs of ribosomal proteins often display severe and distinguishable phenotypes [27,28]. Possibly, the differential composition of ribosomes in response to changes in growth conditions contributes to the selective translation of mRNA subsets [29,30]. Interestingly, sucrose treatment was shown to significantly change ribosomal protein composition [31]. The finding that sucrose concentrations correlate with general translational activity [1] and affect ribosomal protein composition suggest a function for sucrose in translational control and, possibly, mRNA selection. A further source of ribosome heterogeneity is post-translational modification of ribosomal proteins. Recently, a number of plant ribosomal proteins and initiation factors (eIFs) were shown to be phosphorylated in response to photosynthetic activity [32,33]. Interestingly, phosphorylation of eIF3C, eIF5A2, and eIF5A3 was found to be dependent on light, but independent of net photosynthesis [33], indicating that light might play a role in translational control independent of the energy aspect. Light was shown to induce translational activity in etiolated seedlings [34], during the diurnal cycle [18], or after an unexpected dark treatment [19]. However, the specific roles of light and sugars in translational control have not been studied so far. Furthermore, while the general effects of energy metabolism on protein synthesis have been described, the effect of sugars on translation of specific mRNAs has not been studied in detail. Until now, most of the studies on translational regulation address stress mediated reduction of translation, and little information is available on the regulation of increase in translation. It is unknown whether translational inhibition and stimulation use the same or different regulatory mechanisms.

We hypothesize that specific genes are translationally stimulated in response to sugar levels. In this study, we analyze the effect of sucrose on seedlings under light or dark conditions in order to uncouple light and sugar effects and to identify mRNAs differentially translated under these conditions. We confirm the general induction of translation in response to increased sucrose concentration in both light and dark treatments. However, polysomal occupancy of a number of genes was found to increase specifically after sucrose treatment in the light. Most of these sucrose responsive mRNAs are poorly translated under the control (no sucrose) conditions and do not change in steady-state abundance after sucrose treatment. Ribosomal protein mRNAs are among those found to be controlled by sucrose at the translational level. Interestingly, the increase in polysomal occupancy is not accompanied by an increase in ribosome density, indicating the involvement of a novel mechanism in translational stimulation in response to sucrose.

## Results

### Metabolic effects of the sugar treatments in different conditions

**Figure 1:**
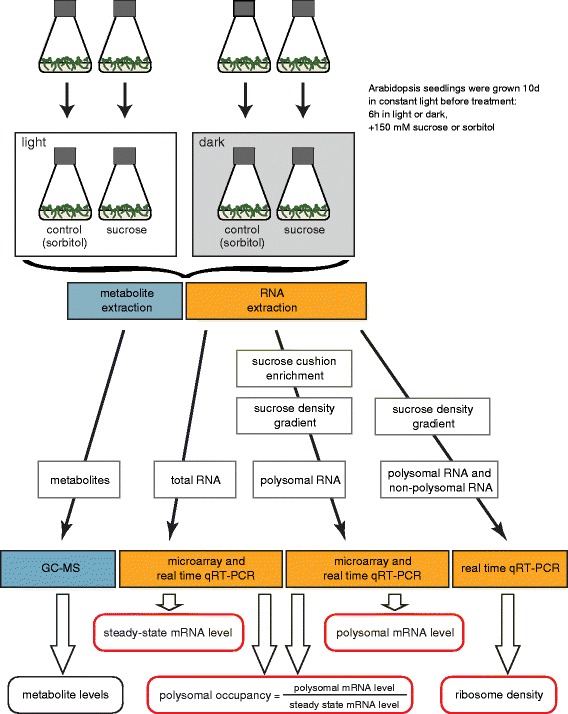
**Schematic summarizing the biological material used and the experiments performed in this study.**

The biological material used and the experiments performed in this study are summarized in Figure 1. Sucrose treatments were applied to Arabidopsis seedlings both in the dark and in the light in order to uncouple the effects of sucrose and light on translation. To adjust for osmotic changes of the media control samples were treated with equimolar concentrations of sorbitol. Metabolite changes induced in the seedlings under the different conditions were analyzed using GC-MS (Table 1, Additional file 1: Table S1). By comparing data obtained for seedlings treated with sorbitol (control) in the dark and in the light, we could conclude that dark control treatment did not induce carbon starvation in our conditions, as sucrose and hexose levels were not significantly affected, similar to most of the identified metabolites. Interestingly, some of the metabolite levels showed a significantly different response to sucrose treatment in the light and in the dark. The levels of amino acids glycine and glutamine increased in the light but not in the dark, while valine and isoleucine decreased in the dark but not in the light. Furthermore, the sucrose-induced increase in hexose and hexose-phosphate levels was stronger in the light. While the organic acids malic acid and citric acid accumulated more in the light than in the dark, succinic acid showed higher levels in the dark. Glycerol-3-phosphate showed reduced levels in the light but not in the dark. This indicates that the sucrose treatment affected metabolism differently in the dark than in the light condition.

**Table 1:** **Changes of metabolite concentrations induced by sucrose treatments in the light and in the dark**

**Metabolites**	**Light sucrose compared to light control**	**Dark control compared to light control**	**Dark sucrose compared to dark control**
***Sugars and related compounds***			
Glycerol-3-phosphate	0.48 *	0.77	0.83
Hexoses	186.7 *	0.94	108.2 ◊
Hexose-6-phosphates	2.34 *	1.27	1.23
Inositol	0.88	1.02	0.86
Sorbitol	0.08 *	0.98	0.05 ◊
Sucrose	53.5 *	1.47	37.4 ◊
***Amino acids and derivatives***			
4-Aminobutyric acid	0.97	1.15	1.21
Alanine	0.96	1.04	0.90
Arginine	1.00	1.17	0.92
Asparagine	1.33	1.21	1.08
Aspartic acid	1.29	0.87	1.20
Glutamic acid	1.47	1.22	1.49
Glutamine	2.06 *	1.12	1.12
Glycine	10.9 *	0.33	2.56
Isoleucine	0.81	1.83 *	0.30 ◊
Lysine	1.06	1.30	0.93
O-acetylserine	1.47	1.35	5.77
Ornithine	1.06	1.28	0.95
Serine	0.41 *	0.82	0.46 ◊
Threonic acid	1.34	1.09	0.90
Threonine	0.87	0.78	0.64
Valine	1.20	1.29	0.69 ◊
***Organic acids***			
4-Hydroxybenzoic acid	0.92	0.98	0.98
Citric acid	7.12 *	0.99	2.26
Fumaric acid	1.54 *	0.69 *	1.38 ◊
Maleic acid	0.89	0.92	0.94
Malic acid	5.67 *	0.56	3.89 ◊
Succinic acid	1.41	0.99	3.70 ◊
***Other compounds***			
Ascorbic acid	1.44 *	0.95	0.98
Gluconic acid	1.13	1.12	0.70 ◊
Octadecanoic acid	0.88	0.97	0.94
Phosphoric acid	0.83	1.33	0.75
Piperidine	0.97	1.10	0.95
Putrescine	1.18	1.17	1.17
t-Sinapinic acid	0.92	1.09	0.97

Values represent the average fold change of different metabolites in treated samples versus the respective control. Asterisks denote a significant difference to the light control (Student’s t-test with Bonferroni correction for multiple testing, p < 0.05), diamond symbol denotes a significant difference to the dark control (Student’s t-test with Bonferroni correction for multiple testing, p < 0.05).

### Sucrose treatments affect transcription differentially in the light than in the dark

Differences in transcript levels induced under the different conditions were studied using microarray analysis (Additional file 2: Table S2). When comparing the sorbitol treated controls, it became apparent that the dark treatment alone affected only few genes (358) in their steady-state level (p < 0.05, more than 2 fold change). 99 of these genes were up-regulated and GO terms related to response to sugar stimuli were found enriched. No genes related to stress response or starvation were induced by the dark control treatment providing further evidence that this treatment does not induce starvation. Among the 259 down-regulated genes, enriched GO terms were related to starch metabolism and the chloroplast, indicating that reduced polysaccharide synthesis and the remobilization of stored reserves might prevent starvation during the dark treatment.

**Figure 2:**
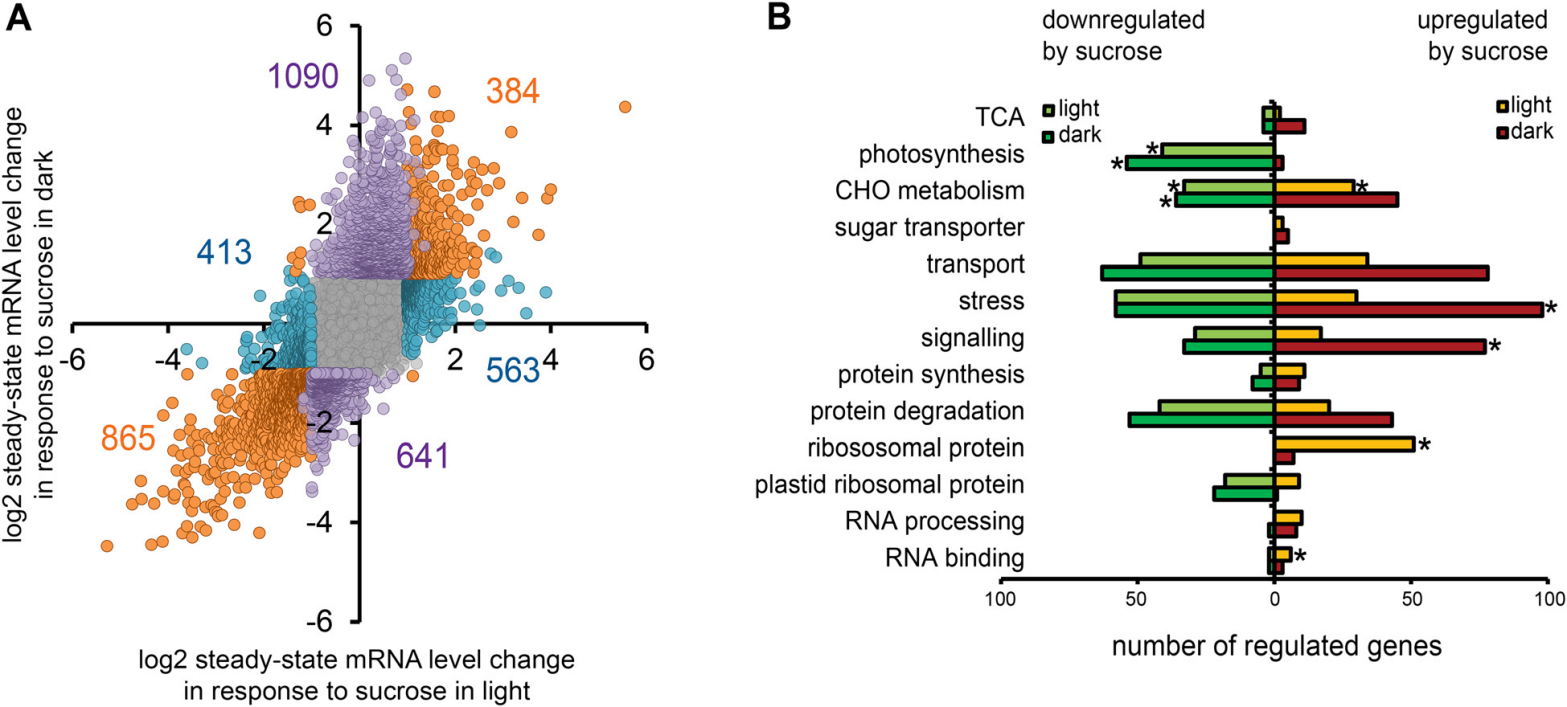
**Transcripts affected by sucrose treatment in Arabidopsis seedlings in the light and in the dark. A)** Plot showing the distribution of expression changes in response to sucrose in the light (x-axis) and dark (y-axis) compared to the corresponding controls. Numbers of genes significantly changed in expression are marked for the different conditions. Purple, blue, and orange dots mark genes affected only in the dark, only in the light, or under both conditions, respectively. **B)** Selected mapman categories of genes up- or down-regulated after sucrose treatment in the light or in the dark. Categories marked with an asterisk contain GO terms found significantly enriched in a topGO analysis. Repressed by sucrose in the light: GO:0015979: photosynthesis (p < 1e-30), GO:0006098: pentose-phosphate shunt (2.2e-20), GO:0019252: starch biosynthetic process (4.7e-11), GO:0000023: maltose metabolic process (5.1e-11). Repressed by sucrose in the dark: GO:0015979: photosynthesis (<1e-30), GO:0006098: pentose-phosphate shunt (1.3e-20), GO:0019252: starch biosynthetic process (6.4e-18),GO:0005984: disaccharide metabolic process (6.5e-10). Induced by sucrose in the light: GO:0006412: translation (2.0e-16), GO:0042254: ribosome biogenesis (6.9e-28), GO:0001510: RNA methylation (<1e-30). Induced by sucrose in the dark: e.g. GO:0010200: response to chitin (<1e-30), GO:0000165: MAPK cascade (<1e-30), GO:0009408: response to heat (<1e-30), GO:0009611: response to wounding (<1e-30), GO:0009723: response to ethylene stimulus (<1e-30).

Sugar treatment induced substantial transcriptional changes in both light and dark. In seedlings treated with sucrose under light condition, the steady state mRNA level of 2225 genes was significantly (p < 0.05, more than 2 fold change) changed, with 947 genes up- and 1278 down-regulated compared to the light control treatment. In seedlings treated with sucrose under dark condition, the expression of 2981 genes was significantly changed, with 1474 genes up- and 1506 down-regulated compared to the dark control. Of the sugar-controlled genes, 967 responded only in the light, and 1728 only in the dark condition, and 1257 were significantly changed in both (Figure 2A, Additional file 3: Figure S1 and Additional file 4: Figure S2 for more comparisons).

The changes observed for steady-state mRNA levels in the different conditions were compared with the measured metabolic changes (Additional file 5: Figure S3). However, no clear pattern was observed that could connect the alterations on the two cellular levels, consistent with studies showing a predominant regulation of metabolism on the post-transcriptional level [35].

GO term enrichment analysis was used to identify significantly enriched functions among the genes affected in the different conditions (Figure 2B). More genes linked to stress and signaling were up-regulated by sucrose treatment in the dark, while genes expressing cytosolic ribosomal proteins were mostly up-regulated in the light. Categories those were down-regulated both in the light and in the dark contained genes involved in photosynthesis and carbohydrate metabolism.

Using the PlantGSEA toolkit for gene set enrichment analysis [36], we found an overlap between previously generated datasets and our microarray results (Additional file 6: Table S3). Genes up-regulated by sucrose treatment [37], carbon fixation [38] or repressed by the KIN10 subunit of SnRK1 [39] overlapped significantly with the list of genes induced by sucrose both in dark and in light in our study. Inversely, genes down-regulated by sucrose treatment in both conditions of our study are to a great extend also repressed by sucrose in another study [37], by carbon fixation [38] or are identified as positively affected by KIN10 [39].

While sucrose-repressed genes showed a great overlap between the dark and the light condition, bigger differences between light and dark were observed for the genes induced by sucrose (Additional file 3: Figure S1 and Additional file 4: Figure S2). In the dark, but not in the light, the sucrose-induced genes overlapped significantly with those found induced by biotic stress response [40,41], in agreement with the enrichment of GO terms linked to stress response and signaling among these genes.

### Sucrose induced translational changes are different in the light and in the dark

**Figure 3:**
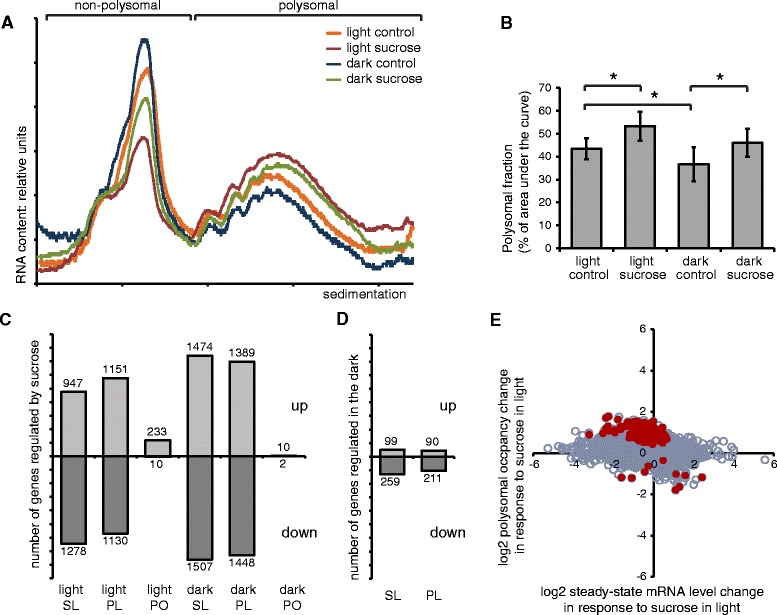
**Effects of sucrose treatment on general and specific translation in the light and in the dark. A)** Absorbance profile of sucrose density gradients. Fractions collected for microarray analysis marked on top. **B)** Polysomal areas as percentage of total area under the curves in different treatments, displayed as difference with the corresponding control. The asterisks mark significant differences (p < 0.05). Average of 14 biological replicates, ±SD. **C)** Number of genes affected by sucrose treatment in the dark or in the light compared to their corresponding control on the transcriptional (steady-state mRNA) level (SL), their polysomal mRNA levels (PL) or their polysomal occupancy (RO, PL/T). **D)** Number of genes affected by dark (sorbitol) treatment compared to the light (sorbitol) control. **E)** Plot showing the polysomal occupancy vs. transcriptional changes induced by sucrose in the light compared to the control. Red dots mark genes with significantly differential ribosomal occupancy.

In order to study to what extent the changes in steady-state mRNA levels are linked to changes in translation of the mRNAs, sucrose density gradient fractionation was performed. General translational activity can be estimated from this data by analyzing the area under the curve in the polysomal fractions (Figure 3A). In the light control samples, 42 ± 5% of the total area was in the polysome fraction. This value dropped to 37 ± 6% in the dark-treated control, while sucrose treatment significantly increased the percentage of polysomal RNA areas to 55 ± 6% in the light and 47 ± 5% in the dark, which corresponds to a difference to the control of 12 ± 4% and 11 ± 6%, respectively (Figure 3B).

In order to analyze the mRNA content by microarray, the polysomal fraction were separated from the non-polysomal fraction (Figure 3A). The polysomal fraction was separated into small and large polysomes, and the mRNA extracted from these fractions was analyzed by microarray analysis. Unexpectedly, an around 90% overlap was observed between the lists of genes affected in the small or large polysome fractions after sucrose treatment. Therefore, the results obtained for the small polysome fractions are discussed here as polysomal mRNA levels in order to simplify the analysis. Comparing the polysomal mRNA levels with changes of steady-state levels allowed identifying alterations in polysomal occupancy, representing the ratio of polysomal mRNA levels to steady-state mRNA levels under the different conditions. The lists obtained for genes altered in their polysomal occupancy in small and large polysomes were fused, as only 4 genes were specific for the large polysomal fraction.

Following sucrose treatment, most mRNAs showed similar changes in steady-state and in polysomal mRNA levels (Figure 3C, Additional file 3: Figure S1 and Additional file 4: Figure S2). Interestingly, polysomal occupancy was changed for a number of genes. We identified 243 genes with altered polysomal occupancy following sucrose treatment in the light and 12 genes affected by sucrose treatment in the dark, with 7 genes found under both conditions. The dark control treatment (compared to the sorbitol light control) affected the polysomal mRNA levels of few genes and none of which was significantly affected in polysomal occupancy in our experimental conditions (Figure 3D).

Sucrose treatment induced the differential polysomal occupancy of 243 mRNAs in the light. Most of these mRNAs were relatively poorly translated in control conditions, whereas sucrose treatment promoted their polysomal mRNA levels. Interestingly, 238 of the 243 genes were detectable in the control samples of a recently published dataset obtained by ribosomal profiling of Arabidopsis seedlings [15], indicating that the genes are translated also in general conditions. For these genes, the translational efficiency under control conditions (1% sucrose) was markedly lower than the average of the whole dataset, indicating that these genes might be translated less than average. Furthermore, only for 31 out of the 243 transcripts the sugar treatment altered steady-state transcript levels significantly (Figure 3E).

A significant higher number, than expected from random distribution of the 243 genes, changed in polysomal occupancy following sucrose treatment in the light could be associated to GO terms related to vesicle-mediated transport, protein degradation, and ribosomal proteins (Additional file 7: Figure S4).

**Figure 4:**
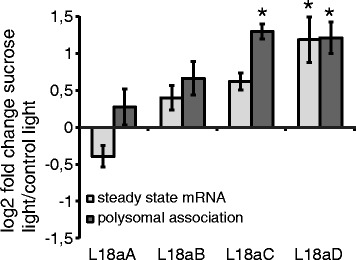
**Effects of sucrose treatments on mRNA abundance and polysomal mRNA levels of the** ***RPL18a*** **family.** Effects of sucrose treatment in the light on steady-state mRNA abundance (light grey) and polysomal mRNA levels (dark grey) of members of the ribosomal protein family RPL18a. Bars show the averages of three biological replicates of log2 fold changes obtained by microarray analysis ± SD. Asterisks mark significant changes in transcript abundance (p < 0.05, log2 fold change >1).

Sucrose treatment in the light affected both steady-state transcript levels and polysome association of ribosomal protein mRNAs. On the microarray, 228 out of the 240 genes encoding ribosomal proteins in *Arabidopsis thaliana* were represented, including *RACK1*, but excluding identified pseudogenes. Of these, steady-state levels of 199 mRNAs were significantly affected, with 44 mRNAs showing an over 2-fold response. Interestingly, the sucrose effect on polysomal mRNA levels of mRNAs encoding ribosomal proteins was more pronounced, with 212 mRNAs significantly affected and an over two-fold change of 116 mRNAs (Additional file 8: Table S4). The ribosomal protein mRNAs significantly affected in their polysomal occupancy belong to 13 of the 81 ribosomal protein gene families. Next to the mRNAs affected in polysomal occupancy, paralogs of all gene families were affected in their steady-state transcription or their polysomal mRNA levels. The RPL18a family, for example, had two paralogs (L18aA and B) not significantly affected in steady-state levels or polysome association, one paralog (L18aD) affected in both, and one paralog (L18aC) affected in polysomal occupancy (Figure 4). For the latter, polysomal mRNA levels were significantly affected, whereas the steady-state mRNA levels remained unaltered. The RPL18a family thus served as an example of different possible regulatory effects that were observed for the different members of a ribosomal protein family.

Interestingly, after sucrose treatment in the dark, 123 ribosomal protein mRNA steady-state levels changed significantly, with 7 mRNAs showing an over two-fold change. Furthermore, 153 mRNAs showed significant changes in polysome association, but only 3 mRNAs showed an over two-fold change. None of the ribosomal protein genes was significantly affected in its polysomal occupancy (Additional file 8: Table S4).

### Ribosome density is decreased after sucrose treatment

**Figure 5:**
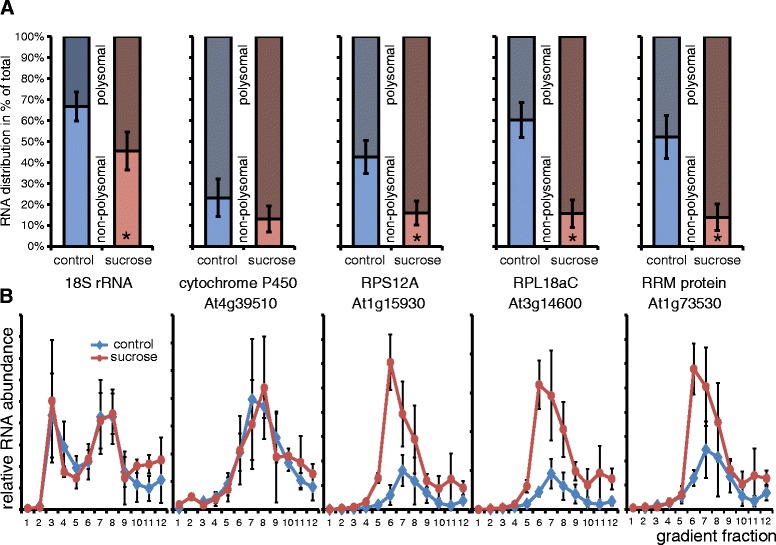
**Real-time qRT-PCR confirmation of the results obtained by microarray analysis for samples treated with sucrose in the light. A)** Comparison of the RNA distribution in non-polysomal (NP, light color) and polysomal (PL, dark color) fractions after performing sucrose gradient fractionation without prior cushion purification. Data was normalized on LUC spike-in mRNA and the sum of signal in NP and PL was set as 100%. Bars show averages of 3 independent biological replicates ± SD. Asterisk denotes significant differences between sucrose and control as determined by Student’s t-test (p < 0.05). **B)** Real-time qRT-PCR analysis of gradient fractions of gradients performed after sucrose cushion enrichment of polysomes from the top (fraction 1) to the bottom of the gradient (fraction 12). Shown are averages of three independent biological replicates ± SD, expression values normalized on LUC spike and on the total area under the curve in the corresponding polysomal gradient analysis.

The distribution of mRNAs between non-polysomal and polysomal fraction was investigated by real time qRT-PCR analysis of mRNA extracted from sucrose density gradient fractions without prior sucrose cushion enrichment. In this way, the total mRNA complement could be studied, including the mRNAs not associated to ribosomes that are removed by the sucrose cushion enrichment. Such experiments allow for the polysomal mRNA levels to be expressed as percentage of total mRNA. Polysomal mRNA association was analyzed for 10 different genes and using a spiked-in luciferase mRNA control for normalization. This experiment confirmed the pattern of polysomal mRNA levels observed in microarray analysis for 5 mRNAs with increased polysomal occupancy (Figure 5A) and 5 with no significant changes in occupancy, as well as the distribution of 18S rRNA (Additional file 9: Figure S5). In this way, the increased polysomal occupancy of mRNAs encoding RPS12A (At1g15930) and RPL18aC (At3g14600) was confirmed, which rose from approx. 30-40% in the control to >80% in the sucrose treated samples (Figure 5A). A similar pattern was observed for an RNA binding protein (At1g73530), but not for a cytochrome P450 family member (At4g39510) as negative control, confirming results obtained in the microarray analysis.

Furthermore, dynamics of the transcripts within the polysomal fraction was studied using real time qRT-PCR analysis of 12 gradient fractions collected from sucrose density gradients loaded with sucrose cushion-enriched samples. Primers for 18S rRNA were used to confirm similar distribution of rRNA in control and sucrose-treated samples after normalization with spiked-in LUC mRNA and the area under the respective curves in the sucrose density gradients (Figure 5B, Additional file 9: Figure S5). Interestingly, the mRNA peaks observed after sucrose treatment appear in a fraction of lower density compared to the control, while a shift to higher density could have been expected considering the increase in polysomal occupancy. Apparently, fewer ribosomes were loaded on a larger fraction of the mRNAs after sucrose treatment. No difference in polysomal occupancy or ribosome density was detected for the cytochrome P450 mRNA (At4g39510) used as negative control.

### Bioinformatic analysis reveals specific characteristics of translationally controlled mRNAs

**Figure 6:**
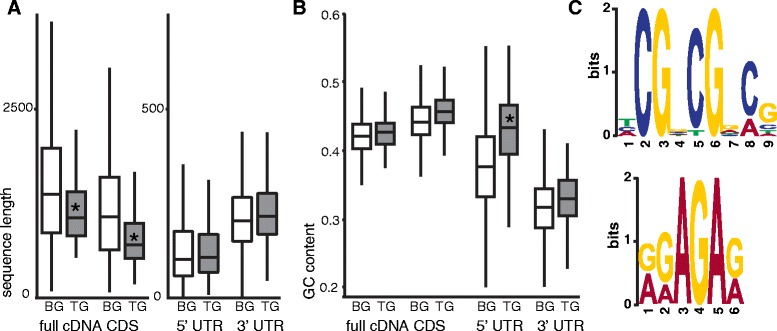
**Analyses of the characteristics linked to the group of genes translationally regulated after sucrose treatment in the light (TG) compared to all genes detected on the microarray (background, BG).** Sequence length **(A)** and GC content **(B)** of the full-length transcript, the coding sequence (CDS) and the untranslated regions (UTR). Asterisk marks differences found significant using Wilcoxon test statistics. Box-and-Whisker plots display median and quartiles, outliers where omitted from the display. **(C)** Two sequence motifs found enriched more than 3-fold in the genes of interest.

Bioinformatic analysis showed mRNA characteristics significantly different in the group of translationally regulated mRNAs compared to the microarray background. The sucrose-regulated mRNAs were significantly shorter in their total mRNA length, as well as in the length of the coding sequence (CDS), whereas the GC content of the 5′UTR was significantly higher (Figure 6A,B). Analysis of the effective number of codons (Nc), which gives an indication of the codon usage bias, could not identify a significant difference between the selected group of genes and the background. The sucrose-regulated mRNAs did not contain more uORFs than the background and their AUGs did not significantly differ from the background consensus. Furthermore, secondary structure analysis did not reveal enrichment in secondary structure.

De-novo motif discovery using MEME identified a number of motifs enriched in the sequences of translationally regulated mRNAs after sucrose treatment (Table 2). Two motifs, [GA][GA]AGA[GA] and [TCA]CG[GCA]CG[GA][CA]G were found enriched more than threefold (Figure 6C).

**Table 2:** **Motifes found enriched in genes regulated on the translational level by sucrose (243 genes) compared to the background (19250 genes) using Fisher’s exact test**

**Consensus**	**Source**	**Translated**	**Background**	**Fold enr.**	**p-value**
[GA][GA]AGA[GA]	5′UTR	29	784	3,2	4,6E-08
G[AG]AGAAGAA[GA]	5′UTR	72	3262	1,9	2,5E-08
GAAGAAG[AC]	CDS	59	2688	1,9	9,2E-07
CG[GA]CG[AG]	5′UTR	82	3413	2,1	1,9E-11
[TCA]CG[GCA]CG[GA][CA]G	5′UTR	55	1476	3,2	7,7E-15
C[TG]TC[TG][TC]C[GT]TC	CDS	160	15406	1,3	3,3E-08
[CT]TCT[TC][TC][CT]TCT	CDS	166	15975	1,4	7,8E-09

## Discussion

Protein synthesis is tightly coordinated with light [19,34] and energy availability [42,43]. In this study, we treated Arabidopsis seedlings with sucrose in the light and in the dark with the aim to uncouple the effect of energy and light availability on the transcription and translation of mRNAs.

Comparing the light and dark control treatments, we concluded that the 6 h dark treatment, in our conditions, did not induce starvation responses. The majority of the measured metabolites, most importantly sucrose and hexoses, did not significantly change in abundance. Furthermore, gene expression changes did not affect stress or starvation related genes. While the dark treatment induced a decrease in polysomal mRNA association, as described before [19], this effect closely followed the gene expression changes and no alteration in polysomal occupancy was detected. Therefore, we used this dark treatment as control for the sucrose treatment in the dark in subsequent experiments, in order to distinguish the sucrose and light specific effects on transcription and translation.

Changes in sugar levels affect the expression of a large number of genes [44]. Sugar treatments repress gene expression related to photosynthesis and energy mobilization, while biogenesis of amino acids, polysaccharides, proteins, and lipids are induced [45,46]. These effects were confirmed here for sucrose treated seedlings under both dark and light conditions. The effect of sucrose treatment on gene expression was larger in the dark than in the light, most evident for sucrose induced genes as compared to sucrose repressed genes. Stress-related genes, for example, were more induced by sucrose in the dark than in the light. Interestingly, most genes encoding cytoplasmic ribosomal proteins showed sucrose-induced expression only in the light. Ribosomal protein genes were found to be regulated by sugar availability in different studies [37–39]. The results presented here suggest that light acts as additional factor necessary for sucrose-induced expression of cytoplasmic ribosomal protein genes. We also show that polysomal occupancy can be enhanced by supplying sugar to non-starved plants.

### Sucrose promotes polysomal occupancy in the light

Polysomal loading was shown to correlate with sucrose concentration in Arabidopsis seedlings [1]. Our results confirmed the general effect of sucrose on translational activity, both in constant light and during dark treatment. In both conditions, the sucrose treatment increased the area under the curve in the polysomal fractions by more than 10% compared to the corresponding control. Many mRNAs were affected in both polysomal mRNA levels and steady-state levels. However, specific changes in only one of these factors were detected for over 450 genes, suggesting translational regulation independent of mRNA levels. More specifically, sucrose treatment caused significantly different polysomal occupancy of 243 mRNAs in light and 12 in the dark, respectively. Thus, sucrose treatment alone is sufficient to induce a general increase in translation concurrent with changes in transcription, whereas light as an additional factor seems necessary to mediate the specific increase of polysomal occupancy.

### Ribosomal protein mRNAs are translationally regulated

Sucrose treatment affected the steady-state levels of 201 mRNAs encoding cytosolic ribosomal proteins, the polysomal mRNA levels of 215, and the polysomal occupancy of 13 mRNAs of the 243 cytosolic ribosomal protein genes present on the microarray. All of the 80 ribosomal protein families are affected on the transcriptional or translational level by sucrose treatment in the light. Interestingly, this regulatory pattern is absent in the dark. This adds a new layer of regulation to the proposed ribosomal protein gene translational ‘regulon’ model stating that ribosomal protein mRNAs are regulated concertedly at the translational level [14,21]. How this translational regulation is achieved remains to be discovered. In mammals, the ribosomal protein mRNAs contain an oligopyrimidine tract in their 5′UTR, the 5′-TOP motif. This motif mediates translational regulation in a mechanism probably involving the TOR kinase [47]. Although this motif has not been observed in plants, the TOR kinase was suggested to be involved in ribosomal biogenesis and the regulation of translation [48–51] and might therefore be involved in the translational regulation described here. Translational regulation could also be achieved by sugar-induced ribosome heterogeneity, as sucrose treatment was shown to affect ribosomal protein composition [31]. Some members of ribosomal protein families where shown to be enriched more than others in ribosomes extracted from sucrose-treated leaves. Furthermore, several ribosomal proteins and compounds of the translational machinery were found differentially phosphorylated dependent on photosynthetic activity [33]. This altered composition might contribute to mRNA selection by the ribosome and thus to their translational regulation [30].

### What is the mechanism of translational stimulation in response to sucrose treatment?

The two parameters, polysomal occupancy and ribosome density, describe the fraction of a certain mRNA being translated and the number of ribosomes per transcript, respectively [14,52]. Using sucrose density gradient centrifugation, we could distinguish these two parameters. Without sucrose cushion enrichment, all ribosome bound and non-bound mRNA will be found in the sucrose density gradient. The shift from the non-polysomal to the polysomal fraction indicates an increase in polysomal occupancy, as more of the total of a transcript is associated with ribosomes [21]. Sucrose cushion enrichment of polysomes removes mRNA not bound to ribosomes. The fractions of the sucrose gradient contain transcripts with increasing numbers of bound ribosomes, thus giving an indication of ribosome density [34]. Fractions obtained using both techniques were analyzed using real time qRT-PCR for 10 mRNAs, of which 5 were affected in their polysomal occupancy on the microarray, and 5 were not.

Polysomal occupancy determined by microarray analysis was confirmed for these 10 mRNAs in independent biological samples. The sucrose induced changes in polysomal occupancy observed by microarray analysis range between 1.3 and 3.4 fold. In the control conditions, the observed polysomal mRNA levels of the mRNAs analyzed by real time qRT-PCR was at least 40%. Therefore, an increase to ~80% corresponds to a two-fold change at most, agreeing with the microarray observations. For the majority of mRNAs, polysomal occupancy is probably close to the optimum in the light control condition and cannot be significantly increased by sucrose addition. Hence, changes in translational status are observed primarily for transcripts that are poorly translated in the control or show a big discrepancy between their transcriptional and translational regulation.

**Figure 7:**
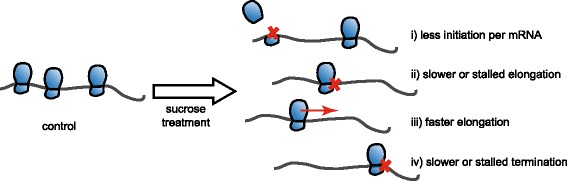
**Schematic model summarizing the possible explanations for the observed pattern in polysomal distribution.**

For the same mRNAs, ribosome density was analyzed in order to get a view on translational dynamics in the different conditions. Interestingly, we observed decreased ribosome density of the 5 transcripts with increased polysomal occupancy. A general effect of the sucrose treatment on ribosome density could be excluded, as the 5 genes with unaltered polysomal occupancy also showed unaltered ribosome density. Changes in initiation frequency, elongation speed, termination, and ribosomal recycling could lead to the observed changes in ribosome density in response to sucrose treatment (Figure 7). Initiation is considered the regulatory step of translation, and increased initiation alone would lead to an increase of both polysomal occupancy and density. However, for the group of mRNAs reported here, we observed increased polysomal occupancy but decreased ribosome density. For these mRNAs, elongation could be slower or even stalled, resulting in decreased initiation per mRNA. However, faster elongation without an increase in initiation could result in the runoff of ribosomes and would present the same shift to lower density fractions. Additionally, termination could be affected and ribosomes stalled on the mRNA. With a concurrent decrease in initiation, this would lead to the observed pattern. Finally, the ribosomal recycling could be affected in a way that could make re-initiation less efficient. Analyzing our biological material using the recently developed technique of ribosomal profiling [15,53,54] would help answering the questions concerning translational dynamics and how translation is affected after sucrose treatment. Furthermore, quantitative proteomic approaches would be necessary to connect the observed changes in polysomal occupancy and density to changes in protein synthesis.

Most of the mRNAs with increased polysomal occupancy upon sucrose treatment in the light seem to be translated less efficiently than average under control conditions. This was confirmed by real time qRT-PCR results on the non-polysomal and polysomal fractions. The genes tested showed no more than 55% of the transcripts in the polysomal fraction, whereas this value was higher for the group of genes not affected in their polysomal occupancy. It was estimated that on average 70% of a transcript is present in polysomes [18], further underlining that the sucrose affected mRNAs were poorly translated in control conditions. The underlying mechanism remains an open question. Comparison with data obtained by immunoprecipitation of UBP1-associated mRNAs that might form granules of inactive transcripts did not identify any of the sucrose controlled genes to be UBP1 associated [16]. It is possible that another regulatory mechanism sequesters these mRNAs in the cytosol and keeps them from being efficiently translated. It remains to be understood how this sequestration might be released or how these mRNAs might be selected for translation after sucrose treatment.

Analysis of the mRNA characteristics of the transcripts affected in their polysomal occupancy by sucrose treatment in the light showed relatively short transcripts and coding sequences, as well as increased GC content in the 5′UTR compared to the background. These mRNA features are suggested to regulate translation under stress conditions [19,55]. Transcripts repressed in translation by dark or hypoxia treatments show a shorter than average CDSs and a higher than average GC content in the 5′UTR. It is possible that the characteristics that lead to a decrease of translation in stress conditions also limit translation efficiency under control conditions. Furthermore, two sequence motifs were found to be enriched in the 5′UTR of these mRNAs. Whereas data on translational regulation by mRNA motifs remains scarce, such motifs might play a role in the sequestration of mRNAs in the cytosol during control condition or in the selection of the mRNAs for translation after sucrose treatment. It is noteworthy that one of the sites identified resembles the well conserved purine-rich motif found in many 5′ leaders of six different families of dicotyledonous plants [56]. However, the motifs were found in a limited number of transcripts and can therefore not explain the mechanism of translational regulation for all the mRNAs affected in their polysomal occupancy after sucrose treatment in the light.

## Conclusions

The translational stimulation in response to sucrose treatment reported here substantiates the diverse reports on plant translational regulation, underlining that several different translational control mechanisms exist in plants. We observed an increase in polysomal occupancy concurrent with decrease in ribosome density in a subset of mRNAs. Enrichment of different motifs and other sequence characters of the regulated mRNAs indicate several regulatory mechanisms operating in parallel. Further studies using both ribosomal profiling as well as specific mutant analyses are required to advance our understanding of these novel regulatory mechanisms.

## Methods

### Biological material

Arabidopsis seeds (var. Col-0) were sterilized in 20% bleach for 20 min, washed 5 times with sterile deionized water, and stratified in water at 4°C for 2 days. Seedlings were grown in 250 ml Erlenmeyer bottles containing 100 ml 0.5 Murashige-Skoog medium for 10 days under constant shaking and in constant light at 22°C. Flasks were covered with aluminum foil or left in the light, and treated with 150 mM sorbitol or sucrose for six hours. Material was harvested by washing seedlings with deionized water and snap-freezing in liquid nitrogen.

### GC-MS metabolic analysis

Metabolite extraction and analysis was performed with six biological replicates as described before [57] with some modifications. The samples (10+/-1 mg each) were extracted using 1 ml chloroform:methanol:H_2_O (1:3:1) supplemented with stable isotope reference compounds for normalization between different samples (7 ng μl^−1^ each of [1,2,3-^13^C_3_]-myristic acid, [1,2,3,4-^13^C_4_]-hexadecanoic acid, [2,2,3,3-^2^H_4_]-succinic acid, [^13^C_5_, ^15^N]-glutamic acid, [25,26,26,26,27,27,27-^2^H_7_]-cholesterol, [^13^C_5_]-proline, [1,2,3,4-^13^C_4_]-disodium 2-oxoglutarate, [^13^C_12_]-sucrose, [2,2,3,3-^2^H_4_]-putrescine, [^2^H_6_]-salicylic acid, and [^13^C_6_]-glucose). Extraction was performed using tungsten carbide beads (3-mm) and a vibration mill (frequency set to 30 Hz) for 3 min. After centrifugation (10 min, 16100 *g*, 4°C), 200 μl of each supernatant was dried in a GC vial using an evacuated centrifuge. For derivatization, 30 μl of methoxyamine hydrochloride (15 mg ml^−1^ in pyridine) were added and the samples were incubated for 16 h at room temperature (RT) after 10 min shaking. Trimethylsilylation was performed with 30 μl of MSTFA (N-Methyltrimethylsilyltrifluoroacetamide) and 1% TMCS (Trimethylchlorosilane) and incubation for 1 h at RT after 1 min shaking. Samples were diluted with 30 μl of heptane containing 15 ng μl^−1^ methylstearate as internal control before analysis by GC-TOFMS (Gas Chromatography–Time of Flight Mass Spectrometer) as described before [57].

Blank control samples and an n-alkanes series (C12-C40) for calculation of retention indices [58] were measured together with the samples. Using an PAL systems auto sampler (Agilent, Atlanta), 1 μl of each sample was injected splitless into an Agilent 7890A gas chromatograph (Agilent, Atlanta) equipped with a 30 m × 0.25 mm (internal diameter) fused silica capillary column with chemically bonded 0.25-μm DB 5-MS stationary phase (Agilent, Atlanta). The injector temperature was 260°C, the septum purge flow rate was 20 ml min^−1^, and the purge was turned on after 75 s. The gas flow rate through the column was 1 ml min^−1^. The column temperature was held at 70°C for 2 min initially before it was increased by 20°C min^−1^ to 320°C and held at 320°C for 14 min. The column effluent was introduced into the ion source of a Pegasus HT TOFMS (Leco Corp., St Joseph, Michigan) using a transfer line temperature of 250°C and an ion source temperature of 200°C. Ions were generated by a -70 eV electron beam at an ionization current of 2.0 mA and 20 spectra s^−1^ were recorded in the mass range 50-800 m z^−1^. The acceleration voltage was turned on after a solvent delay of 290 s. The detector voltage was 1600 V.

Non-processed MS files were exported into MATLAB 8.2 (R2013b; Math Works Natick, Massachusetts), in which all data pretreatment procedures (baseline correction, chromatogram alignment, and hierarchical multivariate curve resolution), were performed using custom scripts [59]. Peak integration and identification by comparing mass spectra and chromatographic retention indices of detected peaks with Umeå Plant Science Center in-house MS library entries and entries of the MS library of the Max Planck Institute in Golm (http://csbdb.mpimp-golm.mpg.de/csbdb/gmd/gmd.html) were done with in-house scripts and NIST MS-Search version 2.0 (NIST, Gaithersburg, MD). Sample peak areas were normalized with the first score vector (t1) derived from a principal component analysis model performed with peak areas of the internal standards reference compounds which were added to the extraction mixture (i.e. all peak areas of one sample were divided by the corresponding t1-value of the respective sample). Additional, sample peak areas were normalized with the sample weights [59].

### Polysome gradients

Ribosomes and ribosome-bound mRNA were extracted and separated over sucrose gradient as described before [60]. Approximately 10 ml of powdered plant material were extracted using 20 ml polysome extraction buffer PEB (0.2 M Tris, pH 9.0, 0.2 M KCl, 0.025 M EGTA, 0.035 M MgCl*2*, 1% Brij-35, 1% Triton X-100, 1% Igepal CA 630, 1% Tween 20, 1% PTE, 5 mM DTT, 50 mg/mL Cycloheximide, 50 mg/mL Chloramphenicol, 80 mM beta-glycerophosphate, 1 mM Sodium Molybdate, protease inhibitor cocktail (Sigma Aldrich), phosphatase inhibitor cocktail 3 (Sigma Aldrich)) and the extract was cleared by filtering through Miracloth™ and centrifugation. An aliquot of 500 μl was taken for total mRNA extraction; the extracts were loaded on top of a sucrose cushion (1.75 M sucrose in PEB without detergents) and centrifuged (18 h, 90000 *g*) in a Beckman Ti70 rotor. The resulting pellet was taken up in wash buffer (0.2 M Tris, pH 9.0, 0.2 M KCl, 0.025 M EGTA, 0.035 M MgCl*2*, 5 mM DTT, 50 mg/mL Cycloheximide, 50 mg/mL Chloramphenicol, 80 mM beta-glycerophosphate, 1 mM Sodium Molybdate) and loaded on a 20-60% sucrose gradient. After ultracentrifugation (1.5 h, 190000 *g*) in a Beckman Sw55Ti rotor, the gradients were fractionated into 12 fractions using a Teledyne Isco Density Gradient Fractionation System with online detection of *A*_254_. These fractions were used separately or pooled into three samples containing non-polysomal material, small polysomes and large polysomes. Areas under the curves were calculated after subtracting the baseline obtained by measuring a blank gradient and normalizing to total area under the curve to account for possible uneven loading of the gradients. When no cushion enrichment step was performed, 1 ml of packed material was extracted with 1 ml of polysome extraction buffer as described above. Extract was filtered and centrifuged before loading of 750 μl directly on 20-60% sucrose gradients. Ultracentrifugation and fractionation were performed as described.

### RNA extraction

RNA was extracted using Guanidine-HCl extraction, followed by a clean-up step using the Spectrum Plant Total RNA Kit (Sigma Aldrich). To each sample (single or pooled fractions), 50 pg of luciferase RNA was added prior to mRNA extraction to allow normalization independent of the original RNA content of the sample.

### Microarray

Microarray analysis was performed in 3 biological replicates using Affymetrix GeneChips Arabidopsis AGRONOMICS Genome Arrays by ServiceXS, Leiden, The Netherlands. Labeled sense stranded cDNA was synthesized with the Ambion WT Expression kit using 100 ng RNA. Fragmentation and terminal labeling with 5.5 μg sscDNA was performed using the Affymetrix Terminal Labeling Kit. Nanodrop and BioAnalyzer were used to assess the concentration and the quality of the cRNA and fragmented sscDNA. 5.5 μg fragmented sscDNA were used for hybridization on the Affymetrix GeneChips Arabidopsis AGRONOMICS Genome Array. Hybridization, washing, and staining were performed with the GeneChip Hybridization, Wash and Stain Kit. Affymetrix GeneChip Command Console (v3.1) software was used to operate the Affymetrix fluidics stations and the scanned array images were analyzed using Affymetrix Command Console Viewer software. The GeneChip data were analyzed using the R statistical programming environment and the Bioconductor packages [61–64]. The aroma.affymetrix package was used to perform RMA background correction, normalization, probe summarization, and quality check of the data. The LIMMA package was used to obtain gene expression data [63]. Steady-state mRNA alterations were calculated using the ratio between the values obtained for total RNA in the sucrose treatment with the corresponding control (light or dark). Polysome association alterations were calculated using the ratio between the values obtained for polysomal RNA in the sucrose treatment with the corresponding control (light or dark). Polysomal occupancy alterations were calculated using the ratio between the polysome association and the steady-state levels. p-values were adjusted for multiple testing with the Benjamini and Hochberg method [61]. GO-term enrichment analysis was performed using the topGO package.

Microarray analysis was performed for three fractions of the sucrose density gradient (non-polysomal, small and large polysome). However, as the non-polysomal fraction contained much less RNA than the others, data obtained for these samples was not used in background correction and normalization to avoid the introduction of artifacts. Furthermore, initial analyses revealed that the list of genes found affected in small and large polysomes were nearly identical. Therefore, data for the small polysomal fraction was used to represent the polysomal fraction. Raw data is deposited along with description of the experimental setup in the GEO repository under the accession nr: GSE59306.

### Real time qRT-PCR

After DNase1 treatment (Thermo Scientific), cDNA was synthesized using the RevertAid Reverse Transcriptase (Thermo Scientific). Quantitative real-time PCR was performed using Power SYBR Green (Applied Biosystems) in a 5 μl reaction using the standard program of a ViiA™ 7 instrument (Applied Biosystems). Data was extracted using ViiA™ 7 Software v1.1. Primer amplification efficiency was calculated using LinRegPCR [65]. Primers used are listed in Additional file 10: Table S5. Results for the primer recognizing the luciferase RNA spike were used to normalize real time qRT-PCR data and the values obtained from gradient fraction samples were reported to the area under the corresponding gradient *A*_254_ curve to adjust for different RNA contents of the samples before extraction.

### Bioinformatic analysis

Both short and long DNA motifs (4-6 and 8-10 nucleotides, respectively) were analyzed in full transcripts, CDS, 5′UTR and 3′UTR (when the corresponding annotation was available) using MEME [66]. Background dinucleotide frequencies were provided separately for each sequence type. To test specificity of the resulting motifs, FIMO [66] was used to scan all genes represented on the microarray for motif hits in the corresponding sequence type. Motifs with FIMO p-value ≤0.001 (short motifs) or ≤0.0001 (longer motifs) were considered significant. Motif enrichment was computed using motif counts for gene lists versus the background using a one-tailed Fisher’s exact test. The distribution of sequence length, codon bias scores [67] and GC content in full transcript, CDS, 5′UTR, and 3′UTR, as well as enrichment for uORFs [68] using Fisher’s exact test, and the analysis of sequence context (-5 to +8) of the uORFs and main ORF start codon were performed using custom scripts comparing the gene list against the background.

## Additional files

Additional file 1: Table S1. Changes in metabolite concentrations induced by sucrose treatments in the light and in the dark. Values represent the relative peak areas observed for the different metabolites. Asterisks denote a significant difference to the light control, diamond symbol denotes a significant difference to the dark control (Student’s t-test with Bonferroni correction for multiple testing, p < 0.05). Additional file 2: Table S2. Microarray data of steady-state mRNA levels, polysomal mRNA levels, and polysomal occupancy for all the genes on the array. Genes with a fold change >2 for their expression values and a corrected p-value <0.05 are marked in the list. Additional file 3: Figure S1. Venn diagrams depicting the overlap of gene lists obtained by data analysis of microarrays. A) sucrose induced steady-state mRNA changes. B) sucrose induced polysomal mRNA levels changes. C) sucrose induced changes of steady-state mRNA levels and polysomal mRNA levels in the light. D) sucrose induced changes of steady-state mRNA levels and polysomal mRNA levels in the dark. E) sucrose induced changes of polysomal occupancy and steady-state mRNA levels in the light. F) polysomal occupancy changes in the dark and in the light. Additional file 4: Figure S2. Plots showing the polysomal association vs. transcriptional changes induced by sucrose in the light (A), in the dark (B), or by dark alone (C) compared to the corresponding control. Yellow points mark genes significantly affected (p < 0.05, more than 2-fold change) in steady state mRNA level, red circles mark genes significantly affected in their polysomal association, and black crosses genes significantly affected (p < 0.05) in their ribosome occupancy. Additional file 5: Figure S3. Mapman analysis of steady-state mRNA changes compared to the changes in metabolite concentrations measured by GC-MS. Changes induced by sucrose in the light A) and in the dark B), as well as by the dark treatment alone C) are displayed on the Mapman output for the TCA cycle. Significantly affected metabolite concentrations are shown using up- and downward pointing arrows next to the depicted metabolite. Additional file 6: Table S3. Summary of PlantGSEA results for lists of genes up- and down-regulated by sucrose treatment in the light or in the dark. Displayed is the overlap of the gene lists with published studies from the database obtained by the standard settings of the website. Additional file 7: Figure S4. Functional analysis of genes found affected in their polysomal occupancy after sucrose treatment in the light. Mapman functional categories found for 154 genes changed in translational loading after sucrose treatment in the light. Not included in the chart were 44 genes without ontology and 45 that fall in miscellaneous groups. Categories marked with an asterisk (*) contained GO terms found significantly enriched in topGO analysis. Additional file 8: Table S4. Changes in steady-state mRNA, polysomal mRNA levels, and polysomal occupancy induced by sucrose in the paralogs of ribosomal proteins. Log-fold changes >1 and p-values <0.05 are marked in color. Additional file 9: Figure S5. Real time qRT-PCR confirmation of the results obtained by microarray analysis for samples treated with sucrose in the light. Bar charts: Comparison of the RNA distribution in non-polysomal (light grey) and polysomal (dark grey) fractions after performing sucrose gradient fractionation without prior cushion purification. Data was normalized on LUC spike-in mRNA and the sum of signal in NP and PL was set as 100%. Bars show averages of 3 independent biological replicates ± SD. Asterisk denotes significant differences between sucrose and control as determined by Student’s t-test (p < 0.05). Line charts: qPCR analysis of gradient fractions of gradients performed after sucrose cushion enrichment of polysomes from the top (fraction 1) to the bottom of the gradient (fraction 12). Shown are averages of three independent biological replicates ± SD, expression values normalized on LUC spike and on the total area under the curve in the corresponding gradient analysis. Additional file 10: Table S5. Primer sequences used for real time qRT-PCR.
