# Supplementary material for: Increased sucrose levels mediate selective mRNA translation in Arabidopsis
Source: BMC Plant Biol. 2014 Nov 18;14:306. doi: 10.1186/s12870-014-0306-3 (PMC4252027; doi:10.1186/s12870-014-0306-3)
Supplement: Additional file 3: Figure S1. — Venn diagrams depicting the overlap of gene lists obtained by data analysis of microarrays. A) sucrose induced steady-state mRNA changes. B) sucrose induced polysomal mRNA levels changes. C) sucrose induced changes of steady-state mRNA levels and polysomal mRNA levels in the light. D) sucrose induced changes of steady-state mRNA levels and polysomal mRNA levels in the dark. E) sucrose induced changes of polysomal occupancy and steady-state mRNA levels in the light. F) polysomal occupancy changes in the dark and in the light. [file 12870_2014_306_MOESM3_ESM.pdf]

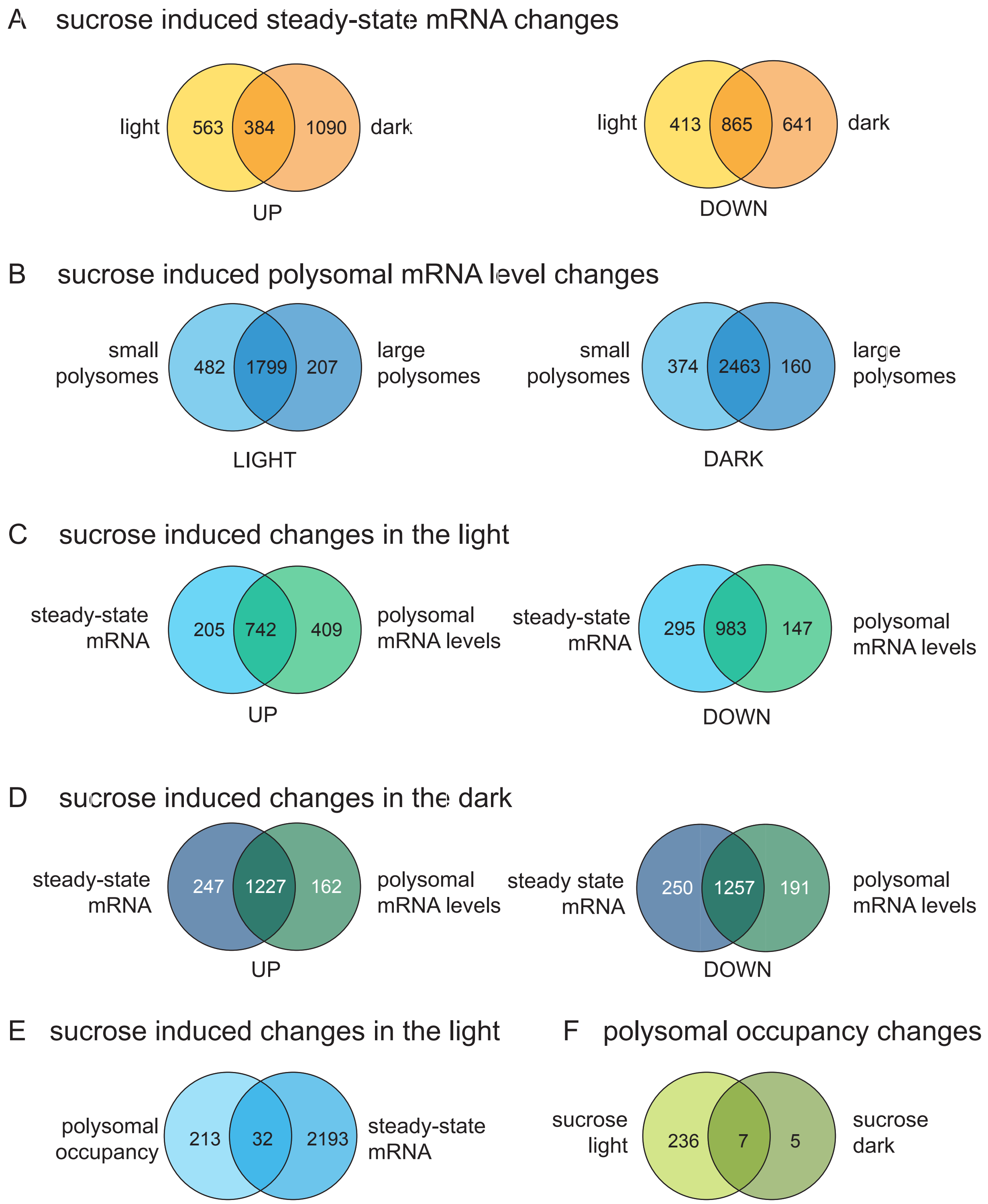

Additional File 3 – Figure S1

Venn diagrams depicting the overlap of gene lists obtained by data analysis of microarrays. A) sucrose induced steady-state mRNA changes. B) sucrose induced polysomal mRNA levels changes. C) sucrose induced changes of steady-state mRNA levels and polysomal mRNA levels in the light. D) sucrose induced changes of steady-state mRNA levels and polysomal mRNA levels in the dark. E) sucrose induced changes of polysomal occupancy and steady-state mRNA levels in the light. F) polysomal occupancy changes in the dark and in the light.
