# Supplementary figures and images for: Increased sucrose levels mediate selective mRNA translation in Arabidopsis
Source: BMC Plant Biol. 2014 Nov 18;14:306. doi: 10.1186/s12870-014-0306-3 (PMC4252027; doi:10.1186/s12870-014-0306-3)

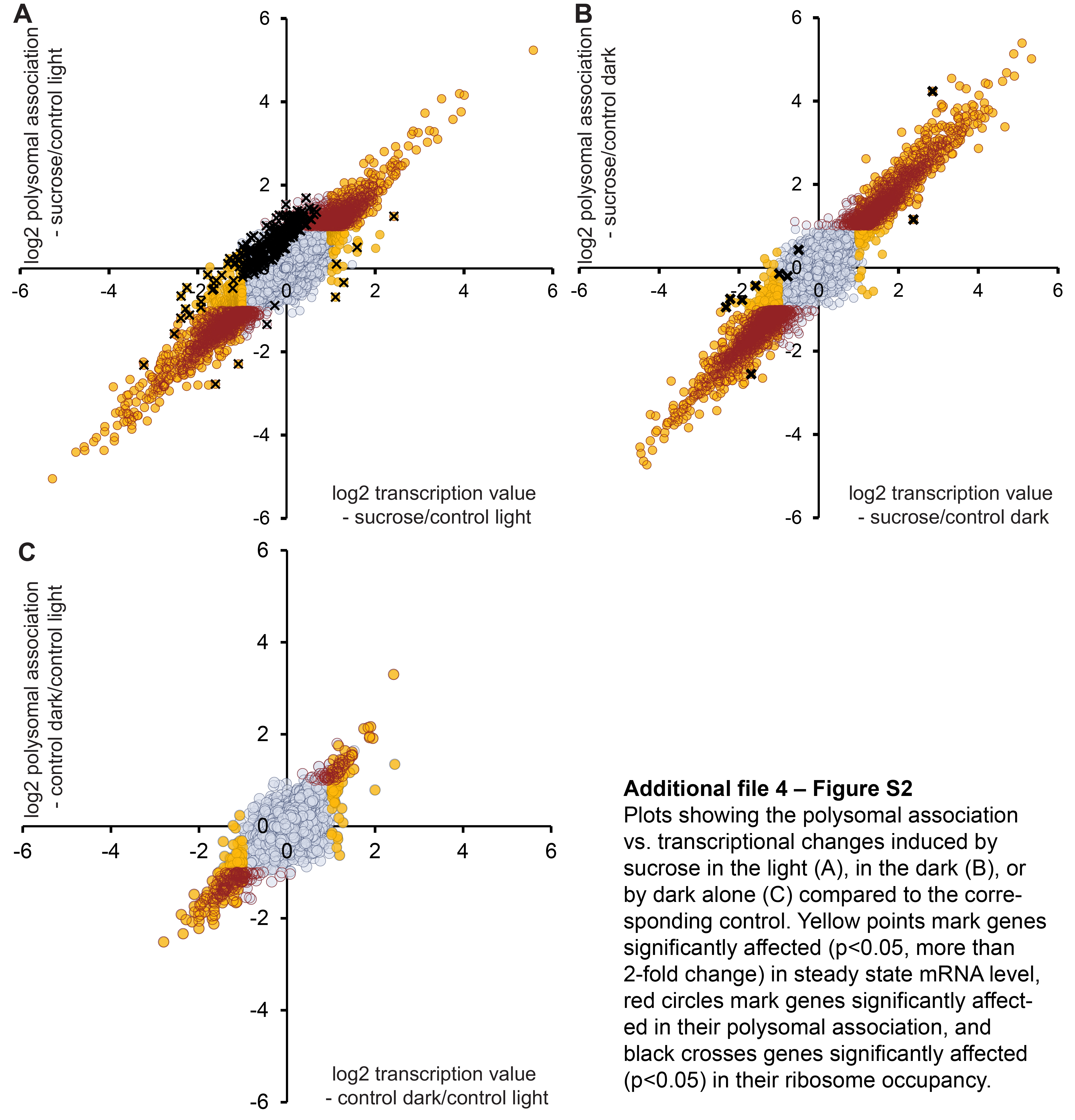

Supplement: Additional file 4: Figure S2. — Plots showing the polysomal association vs. transcriptional changes induced by sucrose in the light (A), in the dark (B), or by dark alone (C) compared to the corresponding control. Yellow points mark genes significantly affected (p < 0.05, more than 2-fold change) in steady state mRNA level, red circles mark genes significantly affected in their polysomal association, and black crosses genes significantly affected (p < 0.05) in their ribosome occupancy. [file 12870_2014_306_MOESM4_ESM.jpeg]
