# Supplementary material for: Increased sucrose levels mediate selective mRNA translation in Arabidopsis
Source: BMC Plant Biol. 2014 Nov 18;14:306. doi: 10.1186/s12870-014-0306-3 (PMC4252027; doi:10.1186/s12870-014-0306-3)
Supplement: Additional file 5: Figure S3. — Mapman analysis of steady-state mRNA changes compared to the changes in metabolite concentrations measured by GC-MS. Changes induced by sucrose in the light A) and in the dark B), as well as by the dark treatment alone C) are displayed on the Mapman output for the TCA cycle. Significantly affected metabolite concentrations are shown using up- and downward pointing arrows next to the depicted metabolite. [file 12870_2014_306_MOESM5_ESM.pdf]

A **sucrose vs control light**

B **sucrose vs control dark**

C **dark control vs light control**

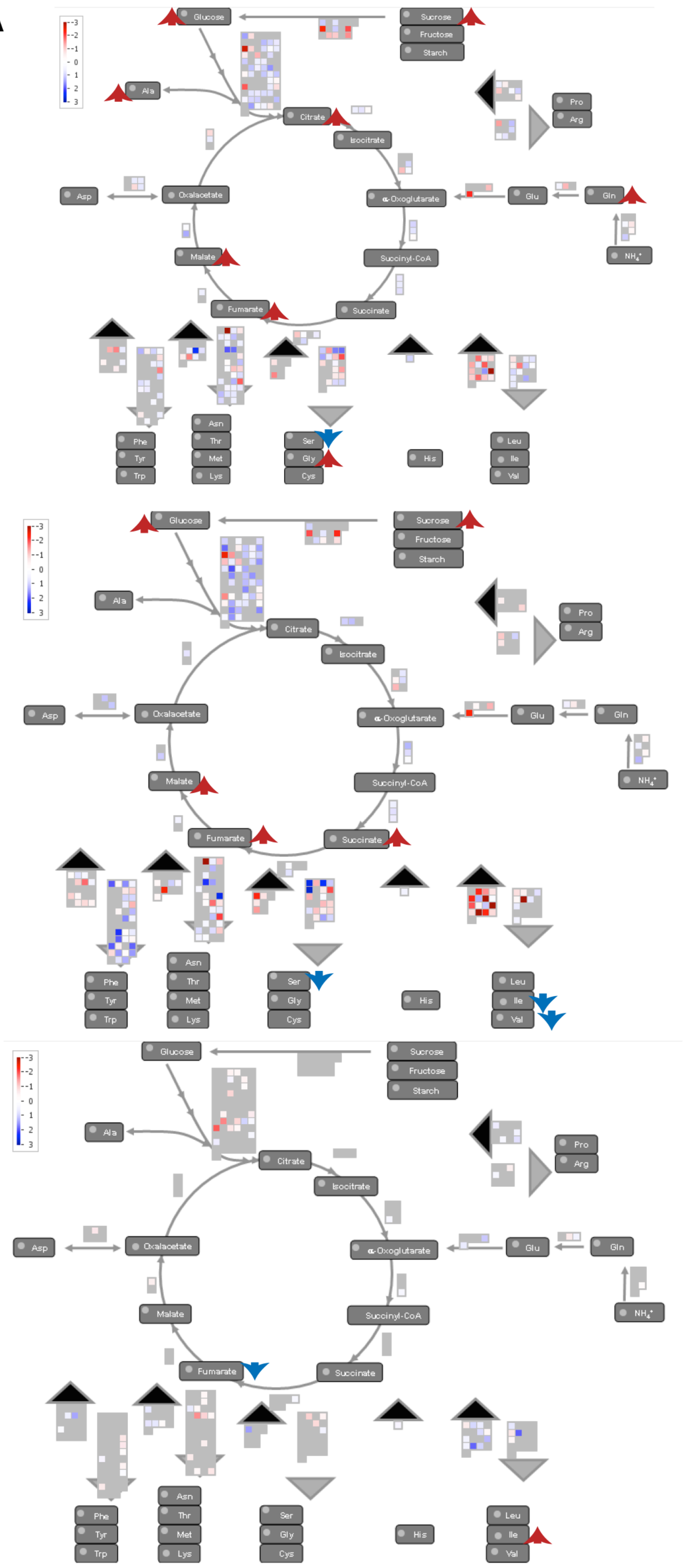

Additional file 5 – Figure S3

Mapman analysis of steady-state mRNA changes in compared to the changes in metabolite concentrations measured by GC-MS. Changes induced by sucrose in the light A) and in the dark B), as well as by the dark treatment alone C) are displayed on the Mapman output for the TCA cycle. Significantly affected metabolite concentrations are shown using up- and downward pointing arrows next to the depicted metabolite.
