# Supplementary material for: Increased sucrose levels mediate selective mRNA translation in Arabidopsis
Source: BMC Plant Biol. 2014 Nov 18;14:306. doi: 10.1186/s12870-014-0306-3 (PMC4252027; doi:10.1186/s12870-014-0306-3)
Supplement: Additional file 7: Figure S4. — Functional analysis of genes found affected in their polysomal occupancy after sucrose treatment in the light. Mapman functional categories found for 154 genes changed in translational loading after sucrose treatment in the light. Not included in the chart were 44 genes without ontology and 45 that fall in miscellaneous groups. Categories marked with an asterisk (*) contained GO terms found significantly enriched in topGO analysis. [file 12870_2014_306_MOESM7_ESM.pdf]

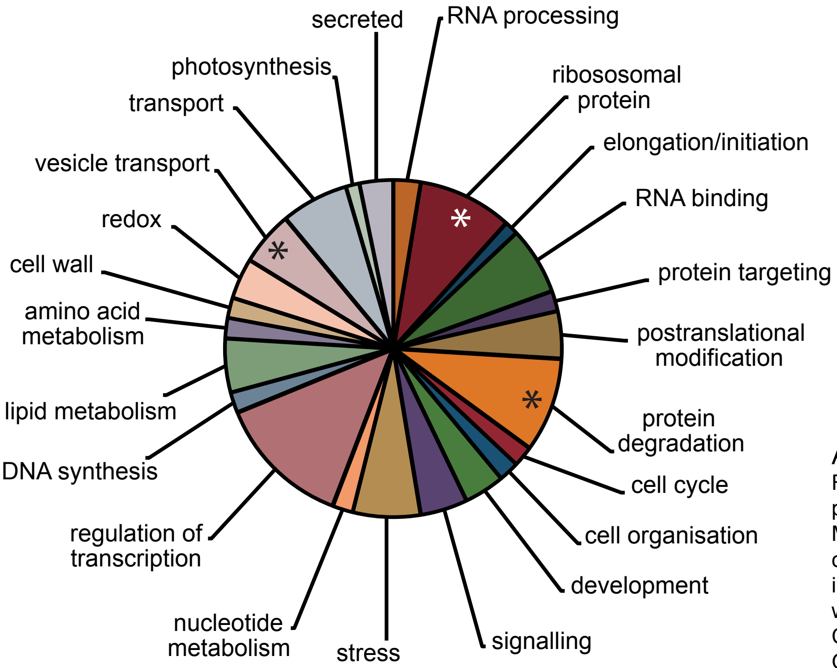

Additional file 7 – Figure S4

Functional analysis of genes found affected in their polysomal occupancy after sucrose treatment in the light. Mapman functional categories found for 154 genes changed in translational loading after sucrose treatment in the light. Not included in the chart were 44 genes without ontology and 45 that fall in miscellaneous groups. Categories marked with an asterisk (*) contained GO terms found significantly enriched in topGO analysis.
