# Supplementary material for: Increased sucrose levels mediate selective mRNA translation in Arabidopsis
Source: BMC Plant Biol. 2014 Nov 18;14:306. doi: 10.1186/s12870-014-0306-3 (PMC4252027; doi:10.1186/s12870-014-0306-3)
Supplement: Additional file 9: Figure S5. — Real time qRT-PCR confirmation of the results obtained by microarray analysis for samples treated with sucrose in the light. Bar charts: Comparison of the RNA distribution in non-polysomal (light grey) and polysomal (dark grey) fractions after performing sucrose gradient fractionation without prior cushion purification. Data was normalized on LUC spike-in mRNA and the sum of signal in NP and PL was set as 100%. Bars show averages of 3 independent biological replicates ± SD. Asterisk denotes significant differences between sucrose and control as determined by Student’s t-test (p < 0.05). Line charts: qPCR analysis of gradient fractions of gradients performed after sucrose cushion enrichment of polysomes from the top (fraction 1) to the bottom of the gradient (fraction 12). Shown are averages of three independent biological replicates ± SD, expression values normalized on LUC spike and on the total area under the curve in the corresponding gradient analysis. [file 12870_2014_306_MOESM9_ESM.pdf]

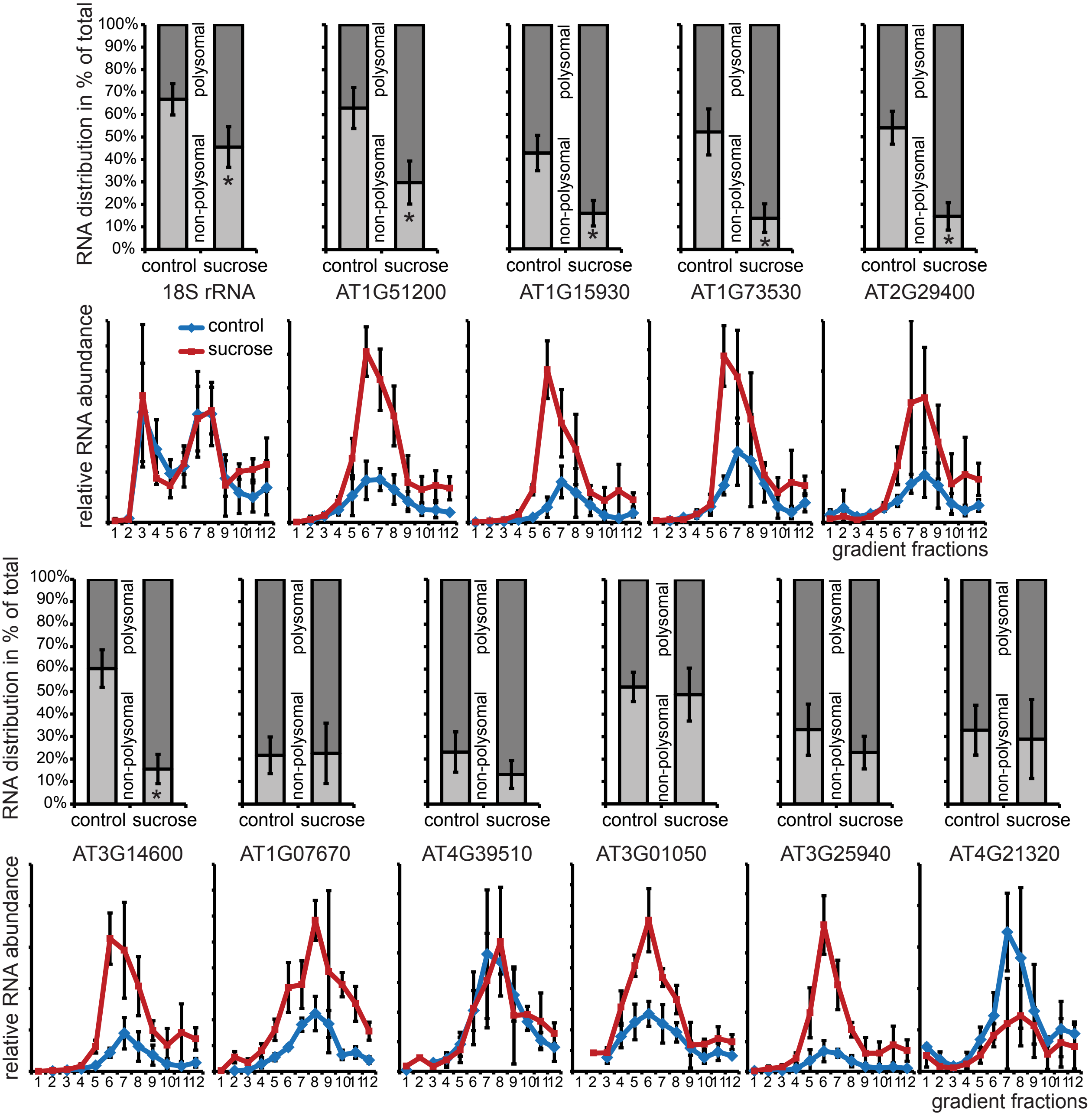

Additional file 9 – Figure S5

Real time qRT-PCR confirmation of the results obtained by microarray analysis for samples treated with sucrose in the light. Bar charts: Comparison of the RNA distribution in non-polysomal (light grey) and polysomal (dark grey) fractions after performing sucrose gradient fractionation without prior cushion purification. Data was normalized on LUC spike-in mRNA and the sum of signal in NP and PL was set as 100%. Bars show averages of 3 independent biological replicates ±SD. Asterisk denotes significant differences between sucrose and control as determined by Student's t-test ($p<0.05$). Line charts: qPCR analysis of gradient fractions of gradients performed after sucrose cushion enrichment of polysomes from the top (fraction 1) to the bottom of the gradient (fraction 12). Shown are averages of three independent biological replicates ± SD, expression values normalized on LUC spike and on the total area under the curve in the corresponding gradient analysis.
